# Impacts of eosinophil percentage on prognosis acute type A aortic dissection patients

**DOI:** 10.1186/s12872-022-02592-y

**Published:** 2022-04-02

**Authors:** Yue Shao, Liu Ye, Hao-ming Shi, Xin-mei Wang, Jun Luo, Lu Liu, Qing-chen Wu

**Affiliations:** 1 Department of Cardiothoracic Surgery, The First Affiliated Hospital of Chongqing Medical University, NO. 1 Youyi Road, Yuzhong District, Chongqing, China; 2 The First Branch, The First Affiliated Hospital of Chongqing Medical University, Chongqing, China; 3 Department of Pathology, Chongqing Medical University, Chongqing, China

**Keywords:** Eosinophil, Acute type A aortic dissection, Mortality, Inflammatory cells, Thrombus, Medical Information Mart for Intensive Care

## Abstract

**Background:**

Eosinophils are pro-inflammatory cells involved in thrombosis and have been proposed as a prognosis marker in acute ischemic stroke and ST-elevation myocardial Infarction. Here, we sought to clarify the prognostic value of eosinophil percentage (EOS%) in patients with acute type A aortic dissection (AAAD).

**Methods:**

We examined 183 consecutive AAAD patients. Based on the optimum cut-off value of EOS% determined by X-tile software, patients were classified into the low EOS% (EOS% ≤ 0.1) and high EOS% groups (EOS% > 0.1). We performed multivariate regression analysis and Kaplan–Meier (KM) survival curves to assess the association between EOS% and mortality. Eosinophil accumulation in aortic dissection intraluminal thrombus was confirmed using hematoxylin–eosin (H&E) staining. An external cohort from Medical Information Mart for Intensive Care IV was performed to validate the results.

**Results:**

Relative to surviving patients, those who died during hospitalization had significantly lower EOS% (*p* = 0.001) but significantly higher WBC (*p* = 0.002) and neutrophil (*p* = 0.001) counts. Multivariate regression analysis identified EOS% as an independent predictor of in-hospital and 1-year mortality. KM curves revealed that 1-year cumulative mortality was significantly higher in the low EOS% group, although it was mainly attributed to the higher 30-day mortality. H&E staining revealed massive infiltration of eosinophils in all 20 thrombus specimens. The external validation confirmed that relative to survivors, patients with in-hospital mortality (*p* = 0.010) had significantly lower EOS%. Moreover, multivariate regression analyses identified that decreased EOS% was independently significantly associated with in-hospital mortality.

**Conclusions:**

Low EOS% is significantly related to increased mortality rates in AAAD patients.

**Supplementary Information:**

The online version contains supplementary material available at 10.1186/s12872-022-02592-y.

## Introduction

Although relatively rare, acute type A aortic dissection (AAAD) is life‐threatening. AAAD in-hospital mortality is about 21.7%. Of these, surgical and medical mortality account for 18.4 and 56.4%, respectively [1], although these may be underestimated. Thus, early identification of individuals at high risk of adverse outcomes is important. Previous findings have demonstrated that C reactive protein (CRP) [2], D-dimer [3, 4], and platelets [5] have been implicated in AAAD mortality. These biomarkers are involved in inflammation or thrombosis in the false lumen [6]. Eosinophils are also involved in various inflammatory responses [7], homeostasis, and thrombosis pathogenesis [8–10], indicating that they are closely associated with aortic dissection (AD) occurrence. Past findings revealed that eosinophil levels in patients with type B AD are significantly lower than those in healthy controls or aneurysms [11, 12]. Nevertheless, the association between eosinophils and AAAD remains to be determined. Here, we investigated whether eosinophil could predict AAAD patients' outcomes.

## Materials and methods

### Study design

This retrospective cohort study involved AAAD patients seen at the First Affiliated Hospital of Chongqing Medical University between September 2014 and July 2020. AAAD diagnosis was confirmed by computed tomography angiography. Exclusion criteria were: (1) age younger than 18 years; (2) missing EOS% data; (3) time of onset > 14 days.

### Data collection and definitions

Baseline information comprised data of age, gender, heart rate (HR), systolic blood pressure (SBP), diastolic blood pressure (DBP), history of smoking, hypertension, Marfan syndrome and surgical procedure. SBP, DBP, and HR were recorded on hospital admission. Laboratory data on admission included, white blood cell (WBC), neutrophil, platelet, lymphocyte counts, monocyte counts, eosinophil percentage (EOS%), platelet–lymphocyte ratio (PLR), Neutrophil–lymphocyte ratio (NLR), lymphocyte-to-monocyte ratio (LMR), procalcitonin, serum albumin, creatine, alanine aminotransferase (ALT), aspartate aminotransferase (AST), urea nitrogen, troponin T (TNT), D-dimer, fibrinogen, and prothrombin time (PT). All cases were followed up at 2 and 4 weeks after discharge and thereafter, every 3 months for at least a year. Primary and secondary endpoints were in-hospital and 1-year mortality, respectively.

### Histopathology

A total of 20 vessel specimens of the aortic arch were taken for histopathological analysis. Aortic fragments were removed during operation and immediately transferred to the laboratory and fixed in 4% paraformaldehyde for 24 h. They were then paraffin-embedded, stained with hematoxylin–eosin (H&E) and examined by an experienced pathologist to identify eosinophil location in the intraluminal thrombus. Images were taken on a microscope (Leica DM2000).

### Statistical analysis

Means (standard deviations) or medians (interquartile ranges) were used to describe continuous variables, which were checked using independent sample t-test or Mann–Whitney U test, respectively. Categorical variables were expressed as counts with percentages and differences between the 2 groups compared using Pearson chi-square test. We performed X-tile software (version 3.6.1) to determine the optimum cutoff of EOS%. Based on the cutoff, we classified patients into two groups. Independent prognostic factors were evaluated using Cox proportional hazards model for 1-year mortality, and logistic regression for in-hospital mortality. Variables with a *p* value < 0.05 in univariate analysis were subjected to multivariate analysis. Results were presented as hazard ratios (HRs) for Cox proportional hazards and odds ratios (ORs) for in-hospital mortality, with their 95% confidence intervals (CIs). Survival probabilities were computed using Kaplan–Meier (KM) method with log-rank test. All statistical analyses were done on R version 3.6.3. *p* < 0.05 was considered statistically significant.

### External validation

External validation was done using MIMIC (medical information mart for intensive care) IV, a large, public database of de-identified patients admitted into critical care units at Beth Israel Deaconess Medical Center from 2008 to 2019. We completed the Protecting Human Research Participants exam to obtain access to this database. This project was granted exemption from ethics by the institutional review boards of Massachusetts Institute of Technology because all data were de-identified. Based on the inclusion and exclusion criteria, 243 patients were enrolled in the analysis. Baseline characteristics are reported in Additional file 1: Table S1.

## Results

### Baseline patient characteristics

Baseline characteristics are listed in Table 1. This study involved 183 AAAD patients (details are shown in Additional file 2: Figure S1). Relative to survivors, patients who died during hospitalization had significantly lower EOS% (*p* = 0.001) but significantly higher WBC (*p* = 0.002) and neutrophil (*p* = 0.001). Compared with survivors, patients with in-hospital death were older and had lower DBP and SBP. Patients who developed adverse outcome had higher levels of NLR, ALT, AST, Scr, urea and TNT, but lower fibrinogen levels. Relative to the non-survivors group, more patients in the survivors group had undergone surgery (72.9% vs. 32.9%, *p* =  < 0.001). Other parameters did not differ significantly. Based on the optimum cut-off value of EOS% determined by X-tile software, patients were classified into the low EOS% (EOS% ≤ 0.1) and high EOS% groups (EOS% > 0.1). Patients with low EOS% had significantly higher white blood cell, neutrophil, PLR, NLR, LMR, AST, d-dimer and PT, while relatively lower fibrinogen and lymphocyte count levels and fewer undergoing surgery (Table 2).

**Table 1:** Baseline characteristics of patients with and without in-hospital mortality

Variables	Survivor	Non-survivor	*p*
	(n = 107)	(n = 76)	
Age (years)	49.53 ± 10.76	53.00 ± 11.74	0.040
Male (n, %)	84 (78.5)	55 (72.4)	0.434
SBP (mmHg)	143.25 ± 29.96	128.28 ± 28.96	0.001
DBP (mmHg)	83.00 (68.50, 94.50)	72.50 (63.00, 87.00)	0.004
HR (bpm)	82.45 ± 15.98	84.03 ± 16.74	0.520
Smoking (n, %)	66 (62.9)	37 (51.4)	0.172
Hypertension (n, %)	70 (65.4)	55 (72.4)	0.404
Marfan syndrome (n, %)	4 (3.7)	3 (3.9)	1.000
White blood cell (× 10^9^/L)	11.68 (9.42, 15.45)	13.50 (11.14, 17.02)	0.002
Neutrophil (× 10^9^/L)	9.83 (7.40, 13.45)	12.17 (9.64, 15.35)	0.001
Platelet (× 10^9^/L)	160.00 (127.25, 196.25)	158.00 (129.25, 192.50)	0.674
Lymphocyte count (× 10^9^/L)	0.95 (0.64, 1.31)	0.82 (0.63, 1.11)	0.201
EOS%	0.10 (0.00, 0.55)	0.10 (0.00, 0.10)	0.001
Monocyte (× 10^9^/L)	0.69 (0.44, 0.88)	0.64 (0.45, 0.98)	0.927
PLR	179.12 (128.44, 254.87)	192.01 (130.65, 255.61)	0.644
NLR	9.9 (5.27, 18.98)	15.28 (9.53, 22.7)	0.005
LMR	1.43 (1.06, 2.06)	1.39 (0.81, 2.09)	0.442
PCT (ng/mL)	0.09 (0.05, 0.27)	0.13 (0.05, 0.47)	0.325
ALB (g/L)	38.18 ± 4.55	37.26 ± 4.21	0.169
ALT (U/L)	30.00 (21.00, 36.50)	33.00 (24.50, 51.00)	0.016
AST (U/L)	24.00 (19.00, 33.00)	31.00 (23.00, 53.50)	0.002
Scr (umol/L)	81.00 (65.50, 100.00)	95.00 (73.50, 119.50)	0.019
Urea (mmol/L)	6.10 (5.05, 7.45)	6.90 (5.45, 8.60)	0.022
Troponin T (ng/mL)	0.01 (0.00, 0.04)	0.02 (0.01, 0.11)	0.014
PT (s)	14.10 (13.40, 14.97)	14.45 (13.60, 15.75)	0.068
Fibrinogen (g/L)	2.70 (2.06, 3.96)	2.20 (1.73, 2.89)	0.004
D-dimer (mg/L)	5.06 (2.50, 10.41)	5.70 (3.41, 11.85)	0.139
Surgery (n, %)	78 (72.9)	25 (32.9)	< 0.001

*SBP* systolic blood pressure, *DBP* diastolic blood pressure, *HR* heart rate, *PLR* platelet–lymphocyte ratio, *NLR* Neutrophil–lymphocyte ratio, *EOS%* eosinophil percentage, *LMR* lymphocyte-to-monocyte ratio, *PCT* procalcitonin, *ALB* serum albumin, *ALT* alanine aminotransferase, *AST* aspartate aminotransferase, *Scr* serum creatine, *PT* prothrombin time

**Table 2:** Baseline characteristics of patients stratified by the optimal cutoff point of EOS% index

Variables	Whole population	EOS% ≤ 0.1	EOS% > 0.1	*p*
	N = 183	N = 124	N = 59	
Age (years)	50.97 ± 11.28	51.90 ± 10.93	49.03 ± 11.84	0.109
Male (n, %)	139 (76.0%)	91 (73.4%)	48 (81.4%)	0.320
SBP (mmHg)	137.03 ± 30.39	137.10 ± 32.10	136.90 ± 26.70	0.967
DBP (mmHg)	78.00 (66.50, 92.00)	77.00 (64.75, 90.25)	82.00 (69.00, 95.50)	0.109
HR (bpm)	83.10 ± 16.27	82.78 ± 16.55	83.78 ± 15.80	0.700
Smoking (n, %)	103 (58.2%)	64 (53.8%)	39 (67.2%)	0.123
Hypertension (n, %)	125 (68.3%)	89 (71.8%)	36 (61.0%)	0.196
Marfan syndrome (n, %)	7 (3.8%)	3 (2.4%)	4 (6.8%)	0.215
White blood cell (× 10^9^/L)	12.61 (10.12, 16.04)	13.66 (11.77, 16.89)	9.52 (7.89, 11.52)	< 0.001
Neutrophil (× 10^9^/L)	10.82 (8.18, 14.18)	12.34 (10.31, 15.31)	7.42 (5.75, 9.29)	< 0.001
Platelet (× 10^9^/L)	160.00 (127.25, 194.00)	157.00 (113.75, 188.00)	169.50 (136.50, 208.00)	0.095
Lymphocyte count (× 10^9^/L)	0.89 (0.64, 1.26)	0.77 (0.60, 0.97)	1.28 (1.00, 1.58)	< 0.001
Monocyte (× 10^9^/L)	0.67 (0.44, 0.95)	0.64 (0.43, 0.93)	0.71 (0.46, 0.96)	0.345
PLR	186.6 (129.66, 255.18)	206.06 (153.94, 282.39)	138.28 (93.93, 193.48)	< 0.001
NLR	12.95 (6.97, 20.14)	16.57 (10.83, 24.17)	5.31 (4.31, 8.09)	< 0.001
LMR	1.43 (0.92, 2.08)	1.29 (0.82, 1.76)	1.82 (1.25, 2.66)	< 0.001
PCT (ng/mL)	0.10 (0.05, 0.31)	0.14 (0.05, 0.43)	0.08 (0.05, 0.18)	0.115
ALB (g/L)	37.80 ± 4.42	38.23 ± 4.11	36.90 ± 4.92	0.057
ALT (U/L)	30.00 (23.00, 42.75)	31.00 (23.50, 45.50)	29.00 (23.00, 36.00)	0.180
AST (U/L)	26.50 (21.00, 38.00)	29.00 (23.00, 41.50)	23.00 (18.00, 33.00)	< 0.001
Scr (umol/L)	85.00 (67.00, 112.50)	87.00 (69.50, 115.00)	81.00 (65.50, 103.00)	0.174
Urea (mmol/L)	6.40 (5.30, 8.18)	6.50 (5.50, 8.25)	5.90 (4.85, 7.20)	0.051
Troponin T (ng/mL)	0.02 (0.01, 0.07)	0.02 (0.01, 0.08)	0.01 (0.01, 0.03)	0.087
PT (s)	14.30 (13.50, 15.23)	14.40 (13.70, 15.50)	13.85 (13.20, 14.90)	0.003
Fibrinogen (g/L)	2.51 (1.95, 3.50)	2.27 (1.72, 2.96)	3.09 (2.34, 4.49)	< 0.001
D-dimer (mg/L)	5.28 (2.96, 10.87)	6.79 (3.41, 13.48)	3.70 (1.84, 6.43)	< 0.001
Surgery (n, %)	103 (56.3%)	60 (48.4%)	43 (72.9%)	0.003

The groups were stratified by the optimal cutoff point of EOS% determined by X-tile software

*SBP* systolic blood pressure, *DBP* diastolic blood pressure, *HR* heart rate, *PLR* platelet–lymphocyte ratio, *NLR* Neutrophil–lymphocyte ratio, *EOS%* eosinophil percentage, *LMR* lymphocyte-to-monocyte ratio, *PCT* procalcitonin, *ALB* serum albumin, *ALT* alanine aminotransferase, *AST* aspartate aminotransferase, *Scr* serum creatine, *PT* prothrombin time

### Relationship between EOS% and mortality

**Fig. 1:**
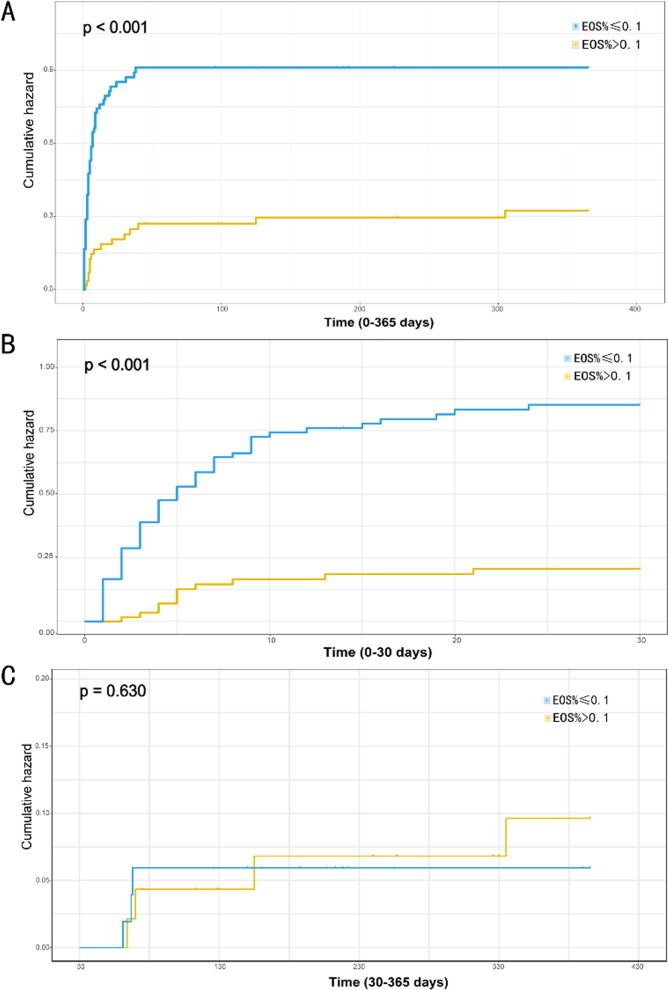
Kaplan–Meier curves for accumulative mortality according to the cut-off of EOS%. **A** Kaplan–Meier curves for 1-year mortality; **B** Kaplan–Meier curves for 30 days mortality. **C** Kaplan–Meier curves for beyond 30 days mortality. *EOS%* eosinophil percentage

We performed logistic regression analyses and Cox proportional hazards models to identify independent predictors of in-hospital and 1-year mortality. Univariate logistic regression analysis identified higher age, low SBP, DBP, EOS%, fibrinogen, high WBC, neutrophil and NLR levels, and non-surgery as important risk factors for in-hospital mortality (Additional file 1: Table S2). Adjusting for these confounders in the multivariate logistic regression analysis, EOS% was independently associated with higher in-hospital mortality (Table 3). Multivariable Cox proportional hazards analysis identified that EOS% was a significant predictor of 1-year mortality (Table 3), independent of variables (including age, SBP, WBC, neutrophils, NLR, ALT, AST, PT, D-dimer, fibrinogen, urea, surgery) that were associated (*p* < 0.05) with outcome in univariate COX regression analysis (Additional file 1: Table S2). KM analysis results on differences in mortality incidence based on EOS% cutoff value are shown in Fig. 1. Patients in the low EOS% (≤ 0.1) group had remarkably higher risks of 1-year mortality than those in the high EOS% (> 0.1) group (Log-rank *p* < 0.001, Fig. 1A). 30-day cumulative incidence of death was significantly higher in the low EOS% group (log-rank *p* < 0.001, Fig. 1B). However, after 30 days, cumulative mortality did not differ significantly between the 2 groups (log-rank, *p* = 0.63, Fig. 1C).

**Table 3:** Predictive value of EOS% for in-hospital and 1-year mortality

Regression models	OR/HR	95% CI	*p*
In-hospital mortality			
Unadjusted model	5.58	(2.68–12.58)	< 0.001
Model I*	5.86	(2.75–13.58)	< 0.001
Model II*	3.25	(1.24–8.87)	0.018
1-year mortality			
Unadjusted model	3.16	(1.84–5.43)	< 0.001
Model I**	3.14	(1.82–5.42)	< 0.001
Model II**	2.48	(1.29–4.79)	0.007

The OR/HR was examined regarding the high EOS% as reference

*OR* odds ratio, *HR* hazard ratio, *95% CI* 95% confidence interval, *EOS%* eosinophil percentage

*Model I: adjusted for age and SBP

*Model II: adjusted for model 1 plus WBC, neutrophils, NLR, fibrinogen, surgery (details shown in Additional file 1: Table S1)

**Model I: adjusted for age and SBP

**Model II: model 1 plus WBC, neutrophils, NLR, ALT, AST, PT, D-dimer, fibrinogen, urea, surgery (details shown in Additional file 1: Table S1)

### Eosinophil infiltration in thrombus specimens

**Fig. 2:**
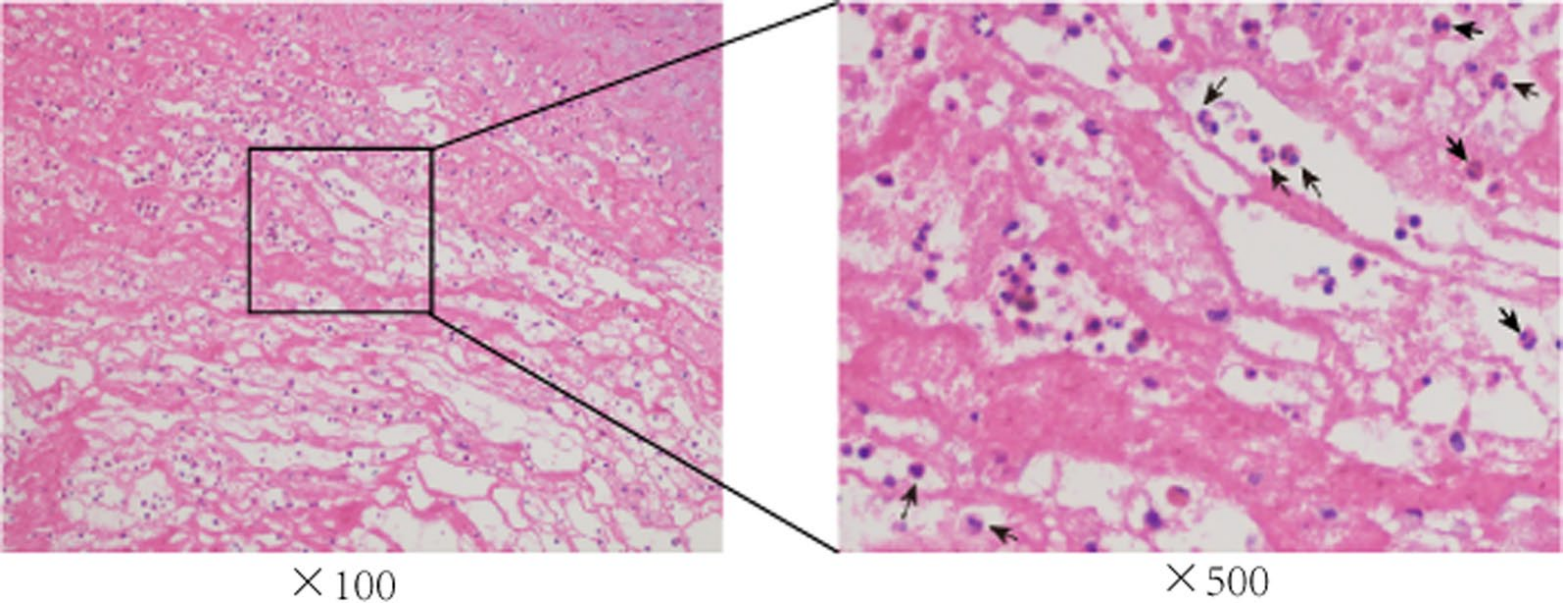
Location of eosinophils within the intraluminal thrombus obtained by surgery. A large number of eosinophils were observed among other inflammatory cells in the thrombus (arrow)

To determine if eosinophils contribute to thrombosis during AD occurrence, we used H&E staining to systematically examine AD intraluminal thrombus and observed the accumulation of eosinophils in all thrombus specimens (Fig. 2).

### External cohort

**Fig. 3:**
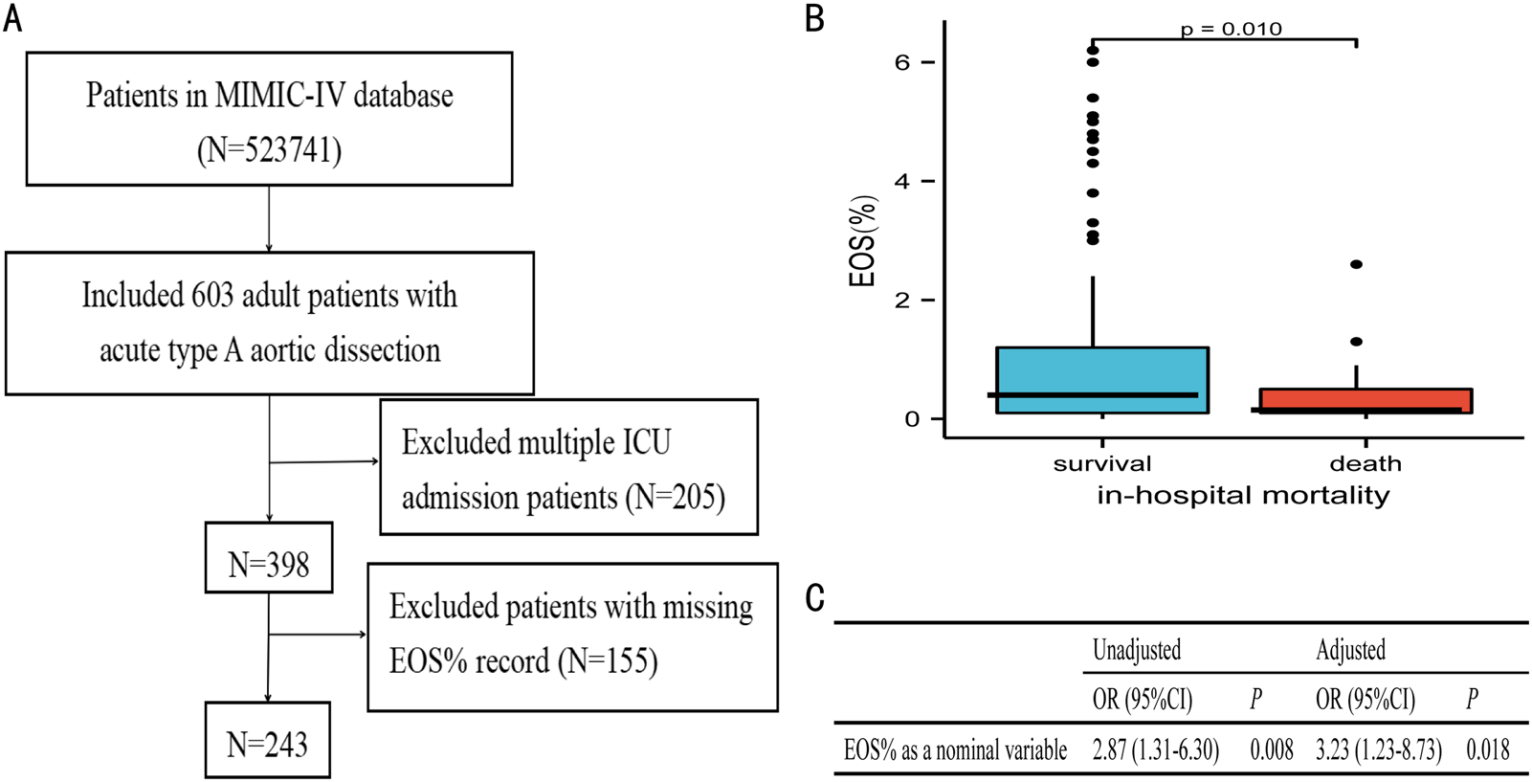
External validation. **A** Flow diagram for patient recruitment. **B** The difference EOS% levels in patients with death or survival groups. **C** Associations of EOS% and in-hospital mortality in the univariate and multivariate logistic regression analyses. Multivariate regression analyses adjusted for hypertension, alb, Scr, urea, surgery (details shown in Additional file 1: Table S3). *MIMIC-IV* Medical Information Mart for Intensive Care IV, *ICU* intensive care unit, *EOS%* eosinophil percentage, *OR* odds ratio, *95% CI* 95% confidence interval

Of 523,741 patients in the MIMIC-IV database, 603 adult patients had been diagnosed with AAAD. Of these, we excluded 205 due to multiple ICU admissions and 155 due to missing data on EOS%, leaving 243 patients that met the inclusion criteria (Fig. 3A). Patients with in-hospital mortality had significantly lower EOS% than survivors (*p* = 0.010, Fig. 3B). Univariate and multivariate regression analyses revealed that decreased EOS% was significantly associated with in-hospital mortality (Fig. 3C).

## Discussion

To our knowledge, this is the first assessment of the predictive value of EOS% on AAAD mortality. Here, we find that: (1) relative to surviving patients, those that died had lower EOS% level and higher WBC and neutrophil counts, (2) despite adjustment for potential confounders, low EOS% was a significant predictor of mortality, (3) Eosinophils accumulated in the aortic dissection thrombus (Additional file 2).

Eosinopenia, first described by Bass et al. in 1980 [13], refers to a marked reduction in the number of circulating eosinophils during acute infection. Eosinophils were subsequently shown to be associated with mortality in critically ill patients [12]. Recently study reported that the relevance of eosinopenia and unfavorable outcomes in patients with acute ischemic stroke [14]. A retrospective study on 606 STEMI patients for 3.5 years suggested that eosinopenia indicates poor cardiac outcomes [15]. Currently, no published studies have evaluated the association between eosinophils and AD and to our knowledge, ours is the first to show that decreased EOS% levels were correlated with in-hospital and 1-year AAAD mortality. Reduced circulating eosinophils increased in-hospital and 1-year mortality by 3–5 and 2–threefold, respectively. This finding was independently verified on the MIMIC IV database, which is comprised of totally different demographic features.

Important inflammatory markers, such as WBCs, neutrophils and prognostic nutritional index, have been found to be associated with poor prognosis in AAAD [5, 16, 17]. Our data show that EOS% negatively correlates with WBC and neutrophil levels, suggesting that there is severe inflammatory reaction when EOS% decreases. Here, multivariate regression analysis displayed reduced EOS% as a vital predictor of AAAD mortality even after adjusting for WBC and neutrophil levels. Moreover, we find that all thrombus samples contain eosinophils, suggesting that eosinophils contribute to thrombus formation and development in AD. This is similar to a previous study by Riegger et al. that eosinophils are present in all stent thrombosis [9]. EOS% seems to be a more suitable predictor of adverse outcomes for AAAD patients because it simultaneously represents inflammation and thrombosis.

There are several potential reasons for the sharp eosinophils decrease in peripheral blood. AAAD, which is associated with severe pain, can evoke acute stress responses that stimulate the release of glucocorticoids like cortisol [18], leading to eosinopenia via apoptosis [19, 20]. Another major reason is that cytokine- and chemokine-mediated eosinophils accumulation at injury sites may reduce circulating eosinophils [21, 22]. On the other hand, aggregated eosinophils in aortic arch involved in development and progression of aortic dissection by regulating inflammatory response and thrombosis. Eosinophils are capable to produce tissue factors [23] and procoagulant phospholipid surface, which can activate prothrombinase complex to generate thrombin, further promoting fibrin formation [8, 24]. Besides, eosinophils interact with platelets at the lesion site leading to mutual activation. Eosinophils migrate into the thrombi and are activated by platelets, thereby promoting the formation of eosinophil extracellular traps (EETs). EETs, which contain major basic protein (MBP), lead to platelet activation by eosinophils. Activated platelets, EETs, and MBP contribute to thrombus formation [25, 26]. Eosinophils are pro-inflammatory cells and can release a great number of cytokines, growth factors, and chemokines, which enhance inflammatory reactions [22, 27, 28]. Cytokines, including interleukin (IL)-1β, IL-2, IL-6, IL-8, and tumor necrosis factor-α, are upregulated in AD patients [28–31], and may promote AD via apoptosis [32, 33]. Eosinophils also express transforming growth factor-β, which is elevated in AD [34] and associated with an upregulation of matrix metalloproteinases [35, 36]. Both are implicated in vascular remodeling via collagen and extracellular matrix degradation [37]. Eosinophils release chemokines like CXC-motif chemokine ligand 8/IL-8, which can recruit leukocytes to the site of inflammation [38].

Some potential limitations should be taken into consideration. Firstly, due to its small sample size and retrospective nature, some bias is inevitable. Secondly, we only confirmed eosinophil presence in AD thrombosis but did not determine how eosinophils promote AD development. Thus, further investigations are warranted to determine the role of eosinophils in AD development.

## Conclusion

Our data identified reduced EOS% as a rapid, simple, and inexpensive tool for predicting AAAD prognosis.

## Supplementary Information

**Additional file 1.** Supplement Table 1.

**Additional file 2.** Supplementary Figure.

## Data Availability

The datasets used and/or analyzed during the current study are available from the corresponding author on reasonable request.
