# Supplementary material for: Impacts of eosinophil percentage on prognosis acute type A aortic dissection patients
Source: BMC Cardiovasc Disord. 2022 Apr 2;22:146. doi: 10.1186/s12872-022-02592-y (PMC8976997; doi:10.1186/s12872-022-02592-y)
Supplement: Supplementary file 1 — Additional file 1. Supplement Table 1. [file 12872_2022_2592_MOESM1_ESM.doc]

Supplementary table 1. Baseline characteristics of patients stratified by the optimal cutoff point of EOS% index in MIMIC IV.

| Variables | Whole population | EOS%≤0.1 | EOS%>0.1 | *P* |
| --- | --- | --- | --- | --- |
|  | N=243 | N=70 | N=173 |  |
| Age (years) | 65.38 ± 13.35 | 64.90 ± 15.06 | 65.58 ± 12.63 | 0.740 |
| Male (n,%) | 133 (54.7%) | 30 (42.9%) | 103 (59.5%) | 0.026 |
| SBP (mmHg) | 130.00 (112.00, 140.00) | 125.50 (110.00, 140.00) | 131.50 (112.25, 142.50) | 0.264 |
| DBP (mmHg) | 79.50 (59.00, 90.00) | 81.00 (61.50, 90.00) | 79.00 (58.25, 90.00) | 0.731 |
| HR (bpm) | 84.00 (70.00, 96.00) | 80.50 (67.25, 91.00) | 85 (72, 99) | 0.182 |
| Hypertension (n,%) | 149 (61.3%) | 43 (61.4%) | 106 (61.3%) | 1.000 |
| Marfan syndrome (n,%) | 2 (0.8%) | 0 (0.0%) | 2 (1.2%) | 1.000 |
| White blood cell (×10^9^/L) | 10.4 (7.55, 13.65) | 12.20 (8.27, 16.65) | 9.80 (7.50, 12.70) | 0.002 |
| Neutrophil (×10^9^/L) | 8.66 (5.82, 11.47) | 9.99 (6.67, 14.43) | 7.92 (5.75, 10.60) | 0.002 |
| Platelet (×10^9^/L) | 154.00 (111.00, 214.00) | 143.50 (113.00, 198.75) | 163.00 (110.00, 219.00) | 0.326 |
| Lymphocyte count (×10^9^/L) | 1.09 (0.74, 1.46) | 0.91 (0.65, 1.29) | 1.14 (0.82, 1.52) | 0.005 |
| Monocyte (×10^9^/L) | 0.40 (0.24, 0.72) | 0.51 (0.24, 0.92) | 0.40 (0.24, 0.68) | 0.105 |
| PLR | 150.31 (90.39, 244.38) | 152.39 (109.79, 290.93) | 149.52 (88.24, 225.74) | 0.179 |
| NLR | 8.14 (4.50, 13.07) | 11.79 (6.46, 15.18) | 7.02 (4.24, 10.56) | < 0.001 |
| LMR | 2.33 (1.28, 4.26) | 1.50 (0.86, 3.11) | 2.63 (1.59, 4.72) | < 0.001 |
| ALB (g/L) | 3.02 ± 0.64 | 2.93 ± 0.60 | 3.06 ± 0.65 | 0.222 |
| ALT (U/L) | 28.00 (16.50, 50.00) | 29.00 (16.00, 55.00) | 27.50 (17.00, 44.25) | 0.740 |
| AST (U/L) | 41.50 (25.00, 84.00) | 41.00 (26.50, 99.00) | 42.00 (25.00, 79.00) | 0.638 |
| Scr (umol/L) | 1.00 (0.80, 1.30) | 1.10 (0.80, 1.30) | 1.00 (0.80, 1.30) | 0.280 |
| Urea (mmol/L) | 18.00 (14.00, 23.00) | 19.00 (14.00, 24.00) | 18.00 (14.00, 23.00) | 0.154 |
| PT (s) | 14.80 (12.80, 18.10) | 15.55 (13.15, 19.80) | 14.50 (12.70, 17.50) | 0.180 |
| Fibrinogen (g/L) | 159.50 (122.00, 223.25) | 159.00 (113.00, 202.00) | 160.00 (123.00, 228.00) | 0.469 |
| Surgery (n,%) | 193 (79.4%) | 54 (77.1%) | 139 (80.3%) | 0.701 |

MIMIC, Medical Information Mart for Intensive Care; SBP, systolic blood pressure; DBP, diastolic blood pressure; HR, heart rate; PLR, platelet–lymphocyte ratio; NLR, Neutrophil–lymphocyte ratio; LMR, lymphocyte-to-monocyte ratio; EOS%, Eosinophil percentage; ALB, serum albumin; ALT, alanine aminotransferase; AST, aspartate aminotransferase; Scr, serum creatine; PT, Prothrombin time.

Supplementary table 2. Univariate regression analyses for in-hospital and 1-year mortality.

|  | In-hospital mortality | | 1-year mortality | |
| --- | --- | --- | --- | --- |
|  | OR (95%CI) | *P* | HR (95%CI) | *P* |
| Age (years) | 1.03 (1.00-1.06) | 0.042 | 1.03 (1.01-1.05) | 0.003 |
| Male/female (n,%) | 0.72 (0.36-1.42) | 0.339 | 0.82 (0.52-1.32) | 0.418 |
| SBP (mmHg) | 0.98 (0.97- 0.99) | 0.001 | 0.99 (0.98-0.99) | 0.003 |
| DBP (mmHg) | 0.98 (0.96-0.99) | 0.003 | 0.99 (0.98-0.99) | 0.026 |
| HR (bpm) | 1.01 (0.99-1.02) | 0.517 | 0.99 (0.99-1.01) | 0.883 |
| Smoking (n,%) | 0.62 (0.34-1.15) | 0.130 | 0.69 (0.45-1.06) | 0.091 |
| Hypertension (n,%) | 1.38 (0.73-2.66) | 0.320 | 1.19 (0.75-1.87) | 0.461 |
| Marfan syndrome (n,%) | 1.06 (0.20-4.94) | 0.942 | 0.84 (0.27-2.67) | 0.772 |
| White blood cell (×10^9^/L) | 1.12 (1.05-1.21) | 0.001 | 1.08 (1.04-1.13) | <0.001 |
| Neutrophil (×10^9^/L) | 1.13 (1.06-1.22) | 0.001 | 1.04 (1.02-1.05) | <0.001 |
| Platelet (×10^9^/L) | 0.99 (0.99-1.00) | 0.409 | 0.99 (0.99-1.00) | 0.204 |
| Lymphocyte count (×10^9^/L) | 0.71 (0.38-1.27) | 0.254 | 0.84 (0.55-1.28) | 0.407 |
| Monocyte (×10^9^/L) | 1.13 (0.51-2.48) | 0.761 | 1.00 (0.57-1.76) | 0.989 |
| PLR | 1.00 (1.00-1.00) | 0.641 | 1.00 (1.00-1.00) | 0.866 |
| NLR | 1.04 (1.01-1.07) | 0.014 | 1.02 (1.01-1.04) | 0.009 |
| LMR | 1.01 (0.82-1.22) | 0.946 | 1.01 (0.87-1.17) | 0.901 |
| EOS% (low *vs* high) | 5.58 (2.68- 12.58) | <0.001 | 3.16 (1.84-5.43) | <0.001 |
| PCT (ng/ml) | 0.93 (0.78-1.01) | 0.267 | 0.93 (0.83-1.04) | 0.226 |
| ALB (g/l) | 0.95 (0.89-1.02) | 0.169 | 0.97 (0.92-1.01) | 0.171 |
| ALT (U/L) | 1.00 (0.99-1.01) | 0.228 | 1.00 (1.00-1.00) | 0.003 |
| AST (U/L) | 1.00 (1.00-1.01) | 0.111 | 1.00 (1.00-1.00) | 0.006 |
| Scr (umol/L) | 1.00 (0.99-1.01) | 0.609 | 1.00 (0.99-1.00) | 0.509 |
| Urea (mmol/L) | 1.03 (0.99-1.08) | 0.135 | 1.01 (1.00-1.03) | 0.027 |
| Troponin T (ng/mL) | 1.22 (0.79-2.11) | 0.387 | 1.07 (0.82-1.39) | 0.636 |
| PT (s) | 1.15 (0.99-1.34) | 0.066 | 1.12 (1.04-1.21) | 0.003 |
| Fibrinogen (g/L) | 0.78 (0.62-0.96) | 0.027 | 0.83 (0.71-0.98) | 0.023 |
| D-dimer (mg/L) | 1.01 (0.99-1.03) | 0.111 | 1.01 (1.00-1.03) | 0.009 |
| Surgery (n,%) | 0.18 (0.09-0.34) | <0.001 | 0.19 (0.12-0.29) | <0.001 |

SBP, systolic blood pressure; DBP, diastolic blood pressure; HR, heart rate; PLR, platelet–lymphocyte ratio; NLR, Neutrophil–lymphocyte ratio; LMR, lymphocyte-to-monocyte ratio; EOS%, Eosinophil percentage; PCT, procalcitonin; ALB, serum albumin; ALT, alanine aminotransferase; AST, aspartate aminotransferase; Scr, serum creatine; PT, Prothrombin time. OR, odds ratio; HR, hazard ratio; 95%CI, 95% confidence interval.

Supplementary table 3. Univariate regression analyses for in-hospital in MIMIC IV.

|  | In-hospital mortality | | | |
| --- | --- | --- | --- | --- |
|  | OR (95%CI) | | *P* |  |
| Age (years) | 1.01 (0.98- 1.04) | 0.460 | | |
| Male/female (n,%) | 1.09 (0.51- 2.40) | 0.820 | | |
| SBP (mmHg) | 1.00 (0.99- 1.02) | 0.605 | | |
| DBP (mmHg) | 1.01 (0.99- 1.03) | 0.421 | | |
| HR (bpm) | 0.99 (0.98- 1.01) | 0.521 | | |
| Hypertension (n,%) | 0.43 (0.20- 0.94) | 0.034 | | |
| Marfan syndrome (n,%) | 0.00 (0.00-0.00) | 0.989 | | |
| White blood cell (×10^9^/L) | 1.05 (0.97- 1.13) | 0.187 | | |
| Neutrophil (×10^9^/L) | 1.05 (0.97- 1.14) | 0.242 | | |
| Platelet (×10^9^/L) | 0.99 (0.99-1.00) | 0.959 | | |
| Lymphocyte count (×10^9^/L) | 1.01 (0.54- 1.76) | 0.974 | | |
| Monocytes (×10^9^/L) | 1.29 (0.62- 2.38) | 0.440 | | |
| PLR | 0.99 (0.99- 1.00) | 0.370 | | |
| NLR | 0.99 (0.95-1.03) | 0.783 | | |
| LMR | 0.98 (0.85- 1.11) | 0.775 | | |
| EOS% (low *vs* high) | 2.87 (1.31- 6.30) | 0.008 | | |
| ALB (g/l) | 0.40 (0.19- 0.80) | 0.012 | | |
| ALT (U/L) | 1.00 (1.00- 1.00) | 0.106 | | |
| AST (U/L) | 1.00 (1.00- 1.00) | 0.069 | | |
| Scr (umol/L) | 1.27 (1.02- 1.56) | 0.025 | | |
| Urea (mmol/L) | 1.02 (1.00- 1.04) | 0.048 | | |
| PT (s) | 1.02 (0.94- 1.08) | 0.633 | | |
| Fibrinogen (g/L) | 1.00 (0.99- 1.00) | 0.853 | | |
| Surgery (n,%) | 0.39 (0.17- 0.90) | 0.023 | | |

MIMIC, Medical Information Mart for Intensive Care; SBP, systolic blood pressure; DBP, diastolic blood pressure; HR, heart rate; PLR, platelet–lymphocyte ratio; NLR, Neutrophil–lymphocyte ratio; LMR, lymphocyte-to-monocyte ratio; EOS%, Eosinophil percentage; ALB, serum albumin; ALT, alanine aminotransferase; AST, aspartate aminotransferase; Scr, serum creatine; PT, Prothrombin time; OR, odds ratio; 95%CI, 95% confidence interval.
