# Supplementary material for: Impacts of eosinophil percentage on prognosis acute type A aortic dissection patients
Source: BMC Cardiovasc Disord. 2022 Apr 2;22:146. doi: 10.1186/s12872-022-02592-y (PMC8976997; doi:10.1186/s12872-022-02592-y)
Supplement: Supplementary file 2 — Additional file 2. Supplementary Figure. [file 12872_2022_2592_MOESM2_ESM.doc]

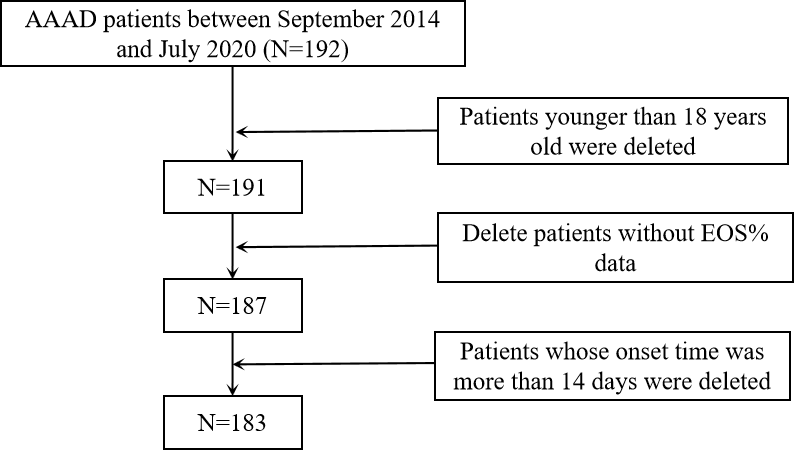


Supplementary Figure 1. Flow diagram for patient recruitment. AAAD, acute type A aortic dissection; EOS%, eosinophil percentage.
